# Effects of Long-Term Regular Continuous and Intermittent Walking on Oxidative Stress, Metabolic Profile, Heart Rate Variability, and Blood Pressure in Older Adults with Hypertension

**DOI:** 10.1155/2022/5942947

**Published:** 2022-01-31

**Authors:** Piyapong Prasertsri, Jatuporn Phoemsapthawee, Sirigoon Kuamsub, Kulwara Poolpol, Orachorn Boonla

**Affiliations:** ^1^Faculty of Allied Health Sciences, Burapha University, Saen Suk, Chonburi 20131, Thailand; ^2^Exercise and Nutrition Innovation and Sciences Research Unit, Burapha University, Saen Suk, Chonburi 20131, Thailand; ^3^Department of Sports Science and Health, Faculty of Sports Science, Kasetsart University, Nakhon Pathom 73140, Thailand; ^4^Faculty of Abhaibhubejhr Thai Traditional Medicine, Burapha University, Saen Suk, Chonburi 20131, Thailand

## Abstract

It is documented that regular exercise is beneficial for improving the antioxidant system, metabolic system, cardiac autonomic function, and blood pressure in those with hypertension. In this regard, low-intensity exercise is recommended for older adults, particularly those with chronic diseases. This study aimed to compare the effects of long-term regular continuous walking with intermittent walking on oxidative stress, metabolic profile, heart rate variability, and blood pressure in older adults with hypertension. Forty-three participants with hypertension aged 60–80 years were randomly divided into the continuous or intermittent walking (CON or INT) groups. Participants in the CON group walked for 30 min, 3 days/week for 12 weeks. Participants in the INT group split 30 min walking into 3 identical sessions punctuated by a 1 min rest after each session, 3 days/week for 12 weeks. Antioxidant and oxidative stress markers, metabolic markers, heart rate variability, and blood pressure were evaluated before and after the exercise program. Glutathione (GSH), GSH to GSH disulfide (GSSG) ratio, and total GSH increased significantly, and GSSG and malondialdehyde decreased significantly in both groups (*p* < 0.05) without significant differences between groups. Triglycerides, ratio of total cholesterol to high-density lipoprotein cholesterol, and atherosclerogenic index were significantly lower in the CON group than those in the INT group (*p* < 0.05). The standard deviation of the NN intervals and root mean square of the successive differences were significantly higher, and low-frequency power was significantly lower in the INT group than that in the CON group (*p* < 0.05). No significant changes in blood pressure were noted in both groups, and nor were there any significant differences between groups. Long-term regular continuous and intermittent walking may comparably increase antioxidants, reduce oxidative stress, and be beneficial for improving important blood pressure-related outcomes, including metabolic profile or cardiac autonomic function in older adults with hypertension.

## 1. Introduction

Hypertension affects more than 1 billion people worldwide, and that number is growing [1], mainly as a result of an aging population [2]. In 2016, 17.9 million or 44% of noncommunicable disease-related deaths were due to cardiovascular disease (CVD) with hypertension the leading risk factor [3]. Hypertension is also the leading preventable risk factor for premature death and disability. Accordingly, treatment and control of hypertension are critically significant for preventing consequent CVD, premature death, and disability [4].

Extensive evidence of cardiovascular, metabolic, and autonomic nervous benefits has led many guidelines to suggest long-term regular exercise as one of the best proven nonpharmacological interventions for preventing and treating hypertension [5]. Long-term regular exercise is also associated with the boost of antioxidant capacity and diminution of oxidative stress levels, which subsequently results in redox balance conservation and cellular homeostasis [6]. Oxidative stress plays a mechanistic role in the control of blood pressure (BP) and the development of hypertension and CVD [6, 7].

Considering exercise for older adults, exercise characteristics, such as intensity, type, and mode, are essential factors that should be judiciously examined. Regarding intensity, low-intensity exercise is often recommended for older adults due to it being easier to perform, which makes it safe and feasible amid practice [8]. Whether an exercise is practiced continuously or intermittently could be another important factor that impacts its effects. Continuous aerobic exercise is defined as any activity using large muscle groups, including walking. Thus, it has been widely reported to produce positive effects on health, including the cardiovascular system [9]. In addition to continuous exercise, intermittent exercise is characterized by repeated short bouts of exercise separated by periods of rest [10], which may be more suited to older individuals with a sedentary lifestyle who are predisposed to a decline in aerobic capacity [11]. In recent years, intermittent exercise has gained a lot of attention for its time-efficient nature and beneficial effects on physical functions [8]. It has been suggested that intermittent exercise results in similar or better physiological outcomes than continuous exercise as a single bout [12]. The earliest studies of intermittent exercise noted that regular practice of this form of exercise is beneficial in terms of modulating vagal activity, adapting the cardiovascular system [13], reducing the risk of metabolic syndrome, and improving body composition [14].

Regarding data from the National Health and Nutrition Examination Surveys (NHANES), Fan and colleagues [15] revealed that the participation rate in walking was ranked first as a leisure time physical activity among all ages of American women. Previous studies reported that the systolic BP (SBP) level of participants with increased BP or essential hypertension reduced significantly by 2.6–21.3 mmHg following regular walking for 12–24 weeks [16, 17]. In rats, walking also correlated positively with metabolic and hemodynamic changes [5]. It is well documented that oxidative stress, cardiovascular risk factors, and cardiac autonomic function are key factors influencing BP [18, 19]. Furthermore, little is known about these responses to low-intensity, continuous, and intermittent walking, particularly as a consequence of long-term practice and in older adults with hypertension. Accordingly, this study aimed to compare the effects of long-term regular continuous walking with intermittent walking primarily on oxidative stress and secondarily on metabolic profile, heart rate variability, and blood pressure in older adults with hypertension.

## 2. Materials and Methods

### 2.1. Study Design and Screening of Participants

This was a pretest-posttest designed experimental study. Fifty older adults in Mueang District, Chonburi Province, Thailand, were recruited from the Aging Society of Mueang District and the Aging Society of Burapha University Hospital, Chonburi Province, Thailand, from February to June 2018. To calculate the sample size, a statistical formula for comparing the mean read-outs of two groups of participants was applied following Hernández-Torres et al. [20]. Continuous and intermittent exercise increased high-density lipoprotein cholesterol (HDLC) to 39.5 and 45.5 mg/dL, respectively, with a standard deviation of 12.7. With an *α* error of 0.05 and a *β* error of 0.20, the proposed sample size of this study was 25 participants per group with a total of 50 participants, including a 10% dropout rate.

Participants were invited to the laboratory and screened on the morning of the physical examination and history taking. The physical examination included BP and heart rate (HR) measurements. In addition, a health questionnaire form and interviews were taken to assess underlying diseases, drug treatment, history of illness, and prior drug treatment. Participants were enrolled in the study after screening, based on the inclusion and exclusion criteria and provision of informed consent. Hypertension was defined by the 2018 ESH/ESC Guidelines [21]. These guidelines classify hypertension as resting SBP of ≥140 mmHg and/or diastolic BP (DBP) of ≥90 mmHg.

The inclusion criteria were (a) male or female; (b) age, 60–80 years; and (c) diagnosed with hypertension or SBP ≥140 mmHg or DBP ≥90 mmHg. The exclusion criteria were (a) obesity, CVD, diabetes mellitus (DM), kidney disease, thyroid disease, or musculoskeletal disease; (b) regular exercise; and (c) regular smokers or drinkers. Consequently, the withdrawal criteria were (a) diagnosed with or received drugs to treat obesity, CVD, DM, kidney disease, thyroid disease, or musculoskeletal disease during participation in the study; (b) unable to practice exercise for at least 80% of the program; and (c) requesting to withdraw from the study.

Most of the participants received BP-lowering medications, such as calcium channel blockers, angiotensin receptor blockers, angiotensin converting enzyme inhibitors, beta-blockers, or diuretics. Eight participants were newly diagnosed and had not received any medications, and 42 were on treatment and reached their target BP (SBP <140 mmHg and DBP <90 mmHg).

This study was conducted under the approval of the Human Ethics Committee of Burapha University (approval no. 218/2560) and was in accordance with the ethical standards of the 2013 Declaration of Helsinki. This study is registered with the Thai Clinical Trials Registry (identification no. TCTR20180226003).

### 2.2. Study Protocols

One week after screening, each participant was invited to the laboratory in the morning to measure the outcomes and receive an exercise program. Each participant was randomized and allocated into continuous or intermittent walking group (CON or INT group). Participants in the CON group were instructed to walk at a rhythm of 1 step/sec or 60 steps/min for 30 min continuously, 3 days/week for 12 weeks. Participants in the INT group were instructed to split 30 min walking into equal 3 sessions with the same rhythm as the CON group, punctuated by a 1 min rest after each session, 3 days/week for 12 weeks.

The exercise program was designed as a home-based exercise. Participants were demonstrated and practiced walking correctly with a metronome obtained from a mobile application (Pro Metronome, EUMLab, Xanin Technology GmbH) under the supervision of a researcher prior to performing the exercise at home. Participants in both groups were asked to maintain their daily dietary intake and routine activities, such as housekeeping, gardening, and cooking without any additional regular exercise, that is, walking for 12 weeks. Participants who could not complete the program (i.e., those exhibiting a health problem or requesting to quit the study) were asked to inform the researcher as soon as possible. Adherence to and compliance with the exercise program were recorded for each participant. Participants were also asked to record their exercise schedule during the study period on the record sheet provided by a researcher. Participants' exercise was closely followed weekly via telephone by a researcher or research assistant. Oxidative stress, metabolic factors, HR variability (HRV), and BP were assessed before and after the exercise program.

### 2.3. Antioxidant and Oxidative Stress Assays

Glutathione (GSH) is an endogenous antioxidant involved in many signaling pathways, including modification of the redox potential toward oxidative values and formation of the disulfide bond between protein thiol groups [22]. GSH, GSH disulfide (GSSG), and total GSH in this study were measured in whole blood as described previously by Nakmareong et al. [23]. The GSH/GSSG redox ratio was obtained by calculation. Malondialdehyde (MDA), a biomarker of lipid peroxidation [24], was determined in plasma using thiobarbituric acid as previously described by Kukongviriyapan et al. [25].

### 2.4. Biochemical Assay

Plasma glucose and serum triglyceride (TG) and total cholesterol (TC) concentrations were measured by enzymatic methods. Serum HDLC concentration was measured with the accelerator selective detergent method. Serum low-density lipoprotein cholesterol (LDLC) concentration was obtained by calculation. These assays were achieved using a standard automated laboratory machine (Architect c8000, Abbott, Lake Bluff, IL, USA). Atherosclerosis is a chronic inflammatory disease of the arteries, associated with blood lipid disorder and oxidative stress [6]. This study also determined the atherosclerogenic index (AI) as an indicator of coronary heart disease and metabolic risk calculated from the following equation: AI = (TC − HDLC)/HDLC.

The participants' venous blood was obtained from an antecubital vein following a 12-hour overnight fast and not taking medications and collected in glucose, EDTA, and clot activator tubes. All analyses were executed within 2 hours after collection.

### 2.5. Blood Pressure and Cardiac Autonomic Function Assessment

Following a 12-hour overnight fast and not taking medications, the participant's BP was measured in the supine position after resting for 15 min, using a digital automatic BP monitor (Microlife BP 3AQ1, Widnau, Switzerland). BP was measured 3 times, 5 min apart, and the average of the three readings was reported. Pulse pressure (PP: SBP − DBP), mean arterial pressure (MAP: DBP + (PP/3)), and rate-pressure product (RPP: SBP × HR) were further calculated from the SBP, DBP, and HR.

HRV, according to a short-term analysis, was used to assess cardiac autonomic function. Lead II electrocardiography (PowerLab 4/30, AD Instruments, Bella Vista, NSW, Australia) was applied to record and analyze the HRV data. Participants' HRV data were collected for 15 min subsequent to the BP measurement. Analysis of HRV data incorporated the time and frequency domains. The time domain consisted of the SDNN and RMSSD values. The frequency domain was comprised of the values of total power (TP), very low, low, and high-frequency powers (VLF: DC to 0.04 Hz, LF: 0.04–0.15 Hz, and HF: 0.15–0.4 Hz), and the LF/HF ratio.

### 2.6. Anthropometry and Body Composition Measurement

Participant height was measured using a stadiometer (Health-O-Meter ProSeries, Pelstar Inc., New York, NY, USA) during inspiration. Fat distribution was measured from waist and hip circumferences and their ratio. Waist circumference (WC) was measured at the end of a normal expiration and at the midpoint between the bottom rib and the superior iliac spine. Hip circumference (HC) was measured on a horizontal plane at the level of maximum buttock extension. Body mass (BM), body mass index (BMI), and body composition (percent body fat, fat mass, fat-free mass, protein mass, mineral mass, and water mass) were measured while wearing minimal clothing using a body composition analyzer (InBody270, InBody Co. Ltd., Deajeon, Korea) based on the principle of bioelectrical impedance analysis. All measurements were taken in the standing position.

### 2.7. Data Analyses

Data normality was analyzed and confirmed using the Shapiro-Wilk test. Two-way repeated measures analysis of variance (with Bonferroni post hoc test) was performed to detect differences in variables between the groups (CON and INT groups) after the exercise program and in each group before and after the exercise program. *F*-values determining whether rejection of the null hypothesis and Cohen's d effect size are also reported in case of having a significant difference between groups. The independent *t*-test was applied to analyze the differences between groups before the exercise program. All analyses were carried out using IBM SPSS Statistics software (IBM Corp., Armonk, NY, USA). Data are shown as mean ± SD. A *p* value <0.05 was considered significant.

## 3. Results and Discussion

Forty-three of 50 participants (86%) completed the study. Seven participants, including 3 participants in the CON group (12%) and 4 participants in the INT group (16%), requested to stop participating in the study as they could not follow the exercise program. Thus, data from 43 participants (22 participants in the CON group and 21 participants in the INT group) were analyzed and reported in the results.

### 3.1. Physical and Physiological Characteristics

No significant differences were observed in physical, except for gender, or physiological characteristics, including age, height, BM, BMI, percent body fat (%BF), fat mass (FM), percent fat-free mass (%FFM), FFM, percent body water (%body water), water mass, protein mass, mineral mass, visceral fat (VF) level, WC, HC, or the W/H ratio between the CON and INT groups before and after the exercise program.

Within-group comparisons demonstrated that HC decreased significantly (*p*=0.047) in the CON group following the exercise program. %BF (*p*=0.026), FM (*p*=0.037), HC (*p*=0.024), and VF level (*p*=0.030) decreased significantly, and %FFM (*p*=0.024) and %body water (*p*=0.026) increased significantly in the INT group following the exercise program (Table 1).

### 3.2. Antioxidant and Oxidative Stress Markers

Antioxidant markers, including blood total GSH, GSH, and GSSG concentrations, the GSH/GSSG ratio, and oxidative stress marker, including plasma MDA concentration, were not different between the CON and INT groups before and after the exercise program.

**Figure 1:**
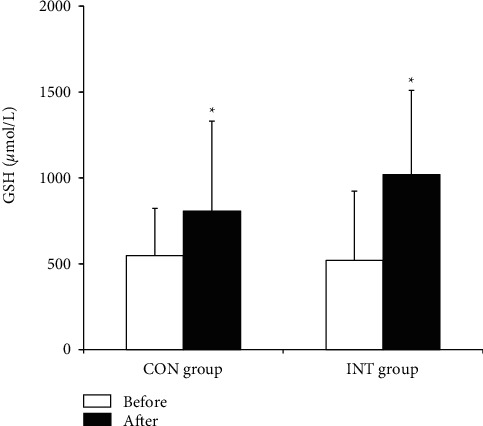
Blood glutathione (GSH) concentration in the continuous and intermittent walking (CON and INT) groups before and after the exercise program. Data are mean ± SD. ^*∗*^*p* < 0.05, significantly different from before the exercise program (two-way ANOVA).

**Figure 2:**
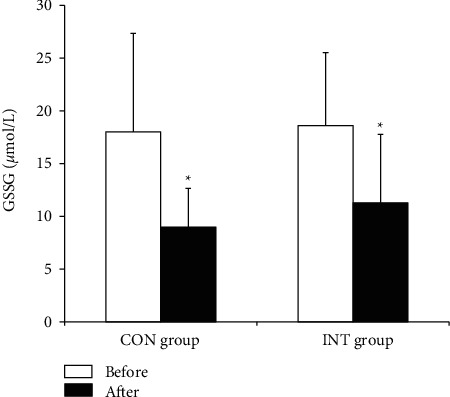
Blood glutathione disulfide (GSSG) concentration in the continuous and intermittent walking (CON and INT) groups before and after the exercise program. Data are mean ± SD. ^*∗*^*p* < 0.05, significantly different from before the exercise program (two-way ANOVA).

**Figure 3:**
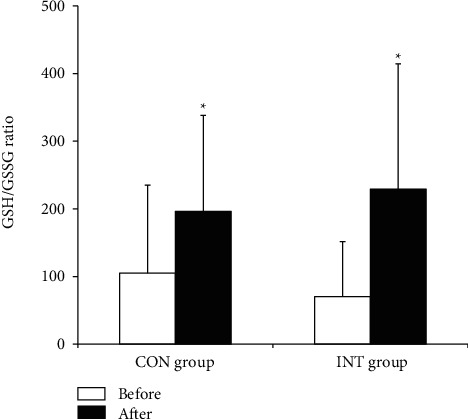
Glutathione to glutathione disulfide (GSH/GSSG) ratio in the continuous and intermittent walking (CON and INT) groups before and after the exercise program. Data are mean ± SD. ^*∗*^*p* < 0.05, significantly different from before the exercise program (two-way ANOVA).

**Figure 4:**
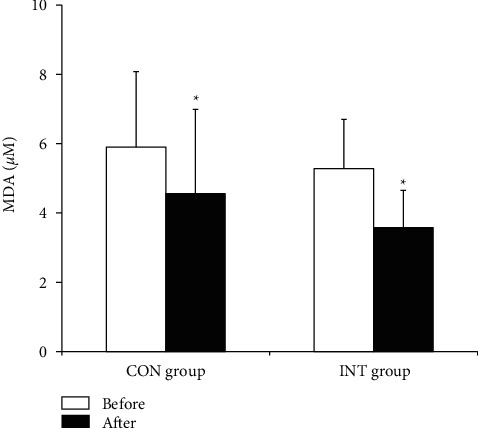
Plasma malondialdehyde (MDA) concentration in the continuous and intermittent walking (CON and INT) groups before and after the exercise program. Data are mean ± SD. ^*∗*^*p* < 0.05, significantly different from before the exercise program (two-way ANOVA).

In both the CON and INT groups, blood total GSH (*p*=0.023 and *p* ≤ 0.001) and GSH (*p*=0.022 and *p* ≤ 0.001) concentrations and the GSH/GSSG ratio (*p*=0.014 and *p*=0.002) increased significantly, and blood GSSG (*p* ≤ 0.001 and *p*=0.002) and plasma MDA (*p*=0.023 and *p* ≤ 0.001) concentrations decreased significantly following the 12-week exercise program (Figures 1–4).

### 3.3. Metabolic Profile

No significant differences were observed in the metabolic variables, including the concentrations of plasma glucose and serum TG, TC, HDLC, and LDLC, the TC/HDLC ratio, and AI between the CON and INT groups before the exercise program. Moreover, TG concentration (*p*=0.017, *F* = 6.224, and effect size = 0.135), the TC/HDLC ratio (*p*=0.048, *F* = 4.151, and effect size = 0.094), and AI (*p*=0.048, *F* = 4.151, and effect size = 0.094) were significantly lower in the CON group than those in the INT group after the exercise program.

HDLC concentration increased significantly (*p*=0.015), and TG concentration (*p*=0.011), the TC/HDLC ratio (*p*=0.003), and AI (*p*=0.003) decreased significantly within the CON group after the exercise program. In addition, plasma glucose concentration decreased significantly (*p*=0.011) within the INT group after the exercise program (Table 2).

### 3.4. Heart Rate and Heart Rate Variability

No significant differences were observed in HR and HRV variables, including SDNN, RMSSD, TP, VLF power, LF power, HF power, or the LF/HF ratio between the groups before the exercise program. Moreover, SDNN (*p*=0.016, *F* = 6.272, and effect size = 0.136) and RMSSD (*p*=0.018, *F* = 6.107, and effect size = 0.132) were significantly higher, and HR (*p*=0.014, *F* = 6.627, and effect size = 0.142) and LF power in the normalized unit (LFnu power) (*p*=0.043, *F* = 4.362, and effect size = 0.098) were significantly lower in the INT group than those in the CON group after the exercise program.

SDNN (*p*=0.028) and RMSSD (*p*=0.047) decreased significantly within the CON group following the exercise program. Besides, HR (*p*=0.006) and LFnu power (*p*=0.006) decreased significantly, and HF power in the normalized unit (HFnu power) closely reached significance (*p*=0.050) within the INT group after the exercise program (Table 3).

### 3.5. Blood Pressure

BP variables, including SBP, DBP, PP, MAP, and RPP, were not different between the groups before the exercise program. However, RPP (*p*=0.049, *F* = 4.123, and effect size = 0.093) was significantly lower in the INT group than that in the CON group after the exercise program.

The BP variables were not different within the CON group, whereas PP (*p*=0.037) and RPP (*p*=0.003) decreased significantly within the INT group following the exercise program (Table 3).

While most hypertension guidelines recommend moderate-to-high-intensity exercise, middle-aged or older adults performing exercise at moderate or high intensity sometimes encounter maladaptation problems, such as exercise-induced joint pain, prolonged palpitation/arrhythmia, chest tightness or dyspnea, and even ischemic events that hinder them from exercising further [26]. Thus, we aimed to explore a low yet effective intensity of exercise to add to the management of hypertension. The primary goal of this study was to establish a practical exercise program that older adults with hypertension could engage in at their leisure with less fatigue and breathlessness, yet provided health benefits. Participants in the two groups walked at a relatively slow speed. They were encouraged to walk at a rhythm of 1 step/sec or 60 steps/min and prescribed a similar 30 min/day volume of exercise. As described by Hall et al. [27] who measured the metabolic cost of walking activities in older adults, the intensity of walking exercise in the present study was classified as low-intensity exercise. Our walking exercise was relatively comparable to slow walking (<3 km/h) at approximately 2.0 metabolic equivalents (METs) [28].

Although walking is defined as low-intensity exercise, this study separated 30 min walking exercise periods into 3 identical sessions (10 min/session), aimed at safer, more feasible practice. In addition, this study included older participants with a sedentary lifestyle (not regular exercisers) exhibiting a decline in aerobic capacity [11]. Thus, partaking in short periods of rest may be more suited to their physical fitness. Moreover, the punctuation of the 10 min walking exercise by a brief 1 min rest (work : rest ratio = 10 : 1) was administered to sustain mitochondrial responses mediated during the 10 min walking [29]. Furthermore, the 1 min rest duration was set concerning participants' fitness level, thereby making this form of walking a suitable option for older individuals with a sedentary lifestyle or with some functional limitations, that is, hypertension, heart diseases, DM, obesity, lung diseases, or musculoskeletal diseases.

### 3.6. Regular Low-Intensity Exercise and Oxidative Stress

To date, studies that have compared continuous to intermittent low-intensity exercise are limited. In this study, long-term regular continuous and intermittent walking was demonstrated to reduce oxidative stress by the increasing number of antioxidant enzymes, thus reinforcing the role of regular low-intensity exercise on the improvement of oxidative stress in physiological and pathological conditions. According to the free radical theory of aging, an accumulation of oxidative damage occurs with aging. This damage is linked to the principal cause of age-related declines in cellular function [30] as well as the development of age-related diseases, including arthritis, DM, dementia, cancer, atherosclerosis, CVD, obesity, osteoporosis, metabolic syndrome, and hypertension [31, 32]. When considering hypertension, chronic inflammation may play a role in the pathogenesis of hypertension by the progression of arterial stiffness [33]. It is involved in high levels of free radicals inducing oxidative stress [34].

It has been proved that regular exercise exerts a vital role in alleviating oxidative stress, improving oxidative metabolism, and reducing inflammation [35]. Regular exercise effectively improves antioxidant capacity in the skeletal muscles, as evidenced by the increased activity of glutathione peroxidase and superoxide dismutase posttraining [36].

The present findings reveal that antioxidants increased significantly and oxidative stress decreased significantly in both hypertensive groups practicing continuous and intermittent walking for 12 weeks. This may reflect the mobilization and synthesis of the antioxidant systems in response to long-term regular exercise. The increase in GSH levels with long-term regular exercise in our study is in congruence with preceding findings [37, 38]. A previously related study by Elokda and Nielsen [38] demonstrated an increased GSH and GSH/GSSG ratio in addition to decreased GSSG levels in healthy sedentary volunteers after regular exercise simulating outpatient cardiac rehabilitation throughout 6 weeks. In adaptation to incremental exercise, Bouzid et al. [39] found that GPx and *α*-tocopherol levels increased significantly in older adults who performed regular low-intensity aerobic exercise.

Mechanisms underlying increased GSH antioxidants after regular exercise, including low-intensity exercise, have previously been described, for instance, upregulation of endogenous antioxidant activity [39, 40]. Besides that, the expression of inflammatory cytokines such as tumor necrosis factor-*α*, which has been revealed to impair intracellular levels of the important cofactor of GPx, is attenuated following regular exercise in chronic heart failure patients [41]. This might be linked to increased concentrations of blood GSH.

In addition to improving antioxidant defense, regular exercise alleviates lipid peroxidation both in adults and in aged individuals [42]. As previously reported in rats, 12 weeks of regular low-intensity cardiovascular exercise reduced oxidative stress and augmented nitric oxide (NO) bioavailability, thus allowing improvement in coronary artery endothelial dysfunction in rats with hypertension [43]. Klarod et al. [44] reported on healthy young adults who performed 8 weeks of regular low-intensity cycling-based exercise, demonstrating that MDA levels decreased significantly. This report further suggested that regular submaximal exercise may prevent lipid peroxidation through lactate metabolism and changes in the NADH/NADPH ratio, which are linked to the tissue adaptive response to exercise. Enhanced lactate uptake through submaximal exercise increases NADH/NADPH ratio, which promotes antioxidant enzyme activity to scavenge free radicals [44].

### 3.7. Regular Low-Intensity Exercise and Heart Rate Variability

The present study also observed an improvement in cardiac autonomic function measured by HRV. HRV is of interest, as a wide range of diseases are associated with decreased variability, including hypertension, DM, CVD, and psychiatric disorders [45]. In this study, SDNN and RMSSD values were significantly greater, and LF power value was significantly lower in participants participating in intermittent walking. These changes were accompanied by a significantly lower HR (−3.4 beats/min). This negative chronotropic effect on HR may be related to an increase in vagal drive as revealed by time domain measures of HRV, that is, an increase in SDNN and RMSDD [46]. SDNN estimates variability due to both sympathetic and parasympathetic contributions and is highly correlated with LF power, especially amid 24 h recordings. The greater SDNN value predicts both lower morbidity and mortality [47]. RMSSD estimates vagally mediated changes reflected in HRV, and the greater RMSSD value is associated with lower scores on a risk inventory of sudden unexplained death [47]. Our results suggest that, with intermittent walking, parasympathetic (vagal) activity may be enhanced, leading to increased HRV, which is associated with the reduced risk of physiological and psychological conditions, that is, DM, CVD, inflammation, obesity, and psychiatric disorders [48]. Our observations are in agreement with a study by Gomes et al. [49] that found 8 weeks of regular low-intensity exercise enhanced cardiac structure and function, decreased oxidative stress, and conserved antioxidant enzyme activity amid rats with aortic stenosis-induced heart failure. These results reveal correlations among oxidative stress, cardiac function, and cardiac autonomic function. In addition, Thiyagarajan et al. [50] explored the association between HRV and elevated oxidative stress in diseases such as hypertension and subsequently found that increased oxidative stress is associated with decreased cardiovagal modulation. Accordingly, it is highly likely that alleviating oxidative stress following regular exercise would lead to improved cardiac autonomic function [51].

### 3.8. Regular Low-Intensity Exercise and Metabolic Profile

Fuller et al. [52] reported that a single session of low-intensity exercise induces rapid and robust shifts in mitochondrial and peroxisomal metabolism in the liver and skeletal muscle of healthy mice. Furthermore, 6 weeks of exercise training at that intensity restricted weight gain, increased muscle glycogen, and decreased TGs, though it did not alter substrate oxidation pathways. A previous study on obese populations by Campbell et al. [53] administered 12 weeks of continuous and intermittent walking exercise and reported that very low-density lipoprotein cholesterol decreased significantly (−0.41 mM/L) over time in the intermittent group, without any significant changes in TC, TG, HDLC, LDLC, and coronary risk ratio in both the continuous and intermittent walking groups. That study suggested that improvements in blood lipids as a result of exercise may be due to greater lipoprotein lipase activity [53]. As compared with that study, our study differed in some particulars, such as age and underlying diseases of participants, exercise frequency, duration, and mode. Hence, our results primarily demonstrate that serum HDLC increased significantly (+3.4 mg/dL). Serum TGs (−23.1 mg/dL), TC/HDLC ratio (−0.4), and AI (−0.4) decreased significantly after continuous walking, and plasma glucose decreased significantly (−5.1 mg/dL) following intermittent walking. Moreover, the intergroup analysis demonstrated that serum TG, TC/HDLC ratio, and AI were significantly lower in participants practicing continuous walking compared with those practicing intermittent walking.

Other related studies on continuous, low-intensity exercise have been reported. Paoli et al. [54] conducted a study on middle-aged overweight men and observed that low-intensity circuit training resulted in decreased TG (−16.2 mg/dL), TC (−6 mg/dL), LDLC (−3.2 mg/dL), and apolipoprotein B/A1 (−0.06). Besides that, there was a greater reduction in SBP (−11 mmHg) compared with high-intensity circuit (−7 mmHg) and endurance training (−5 mmHg). In addition, Gaesser and Rich [55] determined the effect of low-intensity cycling exercise over 18 weeks in healthy males and discovered a significant reduction in %BF, amounting to an average fat loss of approximately 1.35 kg. However, that study did not observe a significant alteration in blood lipids (i.e., TC, TG, HDLC, LDLC, TC/HDLC, and HDLC/LDLC). Teodoro et al. [56] showed that 8 weeks of low-intensity cardiovascular exercise increased antioxidant enzyme activities, decreased lipid hydroperoxide and protein carbonyl formation, and decreased the number of atherosclerotic lesions in mice with atherosclerosis. This study emphasized the links between oxidative stress, metabolic abnormality, and the development of atherosclerosis.

A study on mild spinal muscular atrophy-like mice, which administered low-intensity running for 10 months, revealed that exercise significantly improved lipid metabolism and glucose homeostasis and enhanced oxygen consumption (VO_2_) efficiency [57]. The beneficial effects of low-intensity exercise that differ from other exercises may be related to the differential use of energetic substrates linked to exercise intensity [58]. Regular low-intensity exercise can shift metabolism from *β*-oxidation toward the use of carbohydrate oxidation. This exercise-induced energy pathway modifies lipid and carbohydrate metabolism. Moreover, regular low-intensity exercise increases the expression of mitochondrial respiratory chain complexes, resulting in enhanced mitochondrial oxidative capacity. It also enhances glucose tolerance and is associated with a decrease in mRNA expression of lipogenic enzymes in fat tissues, hence establishing enhanced whole-body metabolism [57].

### 3.9. Regular Low-Intensity Exercise and Body Composition

Results of Going et al. [59] demonstrated a strong relationship between %BF and chronic disease risk factors, that is, BP, lipids and lipoproteins, glucose, insulin, and C-reactive protein levels. %BF has also been shown to be a risk factor for CVD and metabolic syndrome in both men and women, independent of BMI [60]. Targeted reduction of %BF may improve cardiovascular outcomes [61]. To our knowledge, few studies have investigated the effects of intermittent, low-intensity exercise, and most studies have incorporated continuous, low-intensity exercise. In this study, we observed significant changes in body composition only in the intermittent walking group, except for a decrease in hip circumference (−1.3 cm) in the continuous walking group. These changes included decreased FM (−4.1 kg), %BF (−5.8%), visceral fat level (−1.5), HC (−5.2 cm), increased %FFM (+5.9%), and %body water (+4.3%), although BMI did not reach a significant decrease (−1.6 kg/m^2^). Our observations are similar, yet with mostly to a greater extent as compared to previous reports. Findings from a study by Chiu et al. [62] revealed that regular low-intensity exercise at 40 to 50% heart rate reserve resulted in significantly decreased BM (−2.9 kg), BMI (−1 kg/m^2^), WC (−5.3 cm), W/H ratio (−0.04), %BF (−1.70%), and FM (−2.6 kg) in sedentary obese college students. Additionally, Suzuki et al. [63] reported on low-intensity cycling exercise at 40% of maximum VO_2_ (VO_2max_) for 12 weeks, which resulted in decreased BMI (−1.9%) and %BF (−2.0%) in sedentary young females. Further study on patients with metabolic syndrome by Dumortier et al. [64] observed that low-intensity endurance exercise for 2 months improved lipid oxidation (+68.5 mg/min) and body composition, that is, FM (−1.6 kg), WC (−3.5 cm), and HC (−2.2 cm). What is more, Lazzer et al. [65] observed that BM (−7.4%), BMI (−7.7%), and FM (−10.1%) significantly decreased in severely obese adolescents performing low-intensity treadmill exercise at 40% VO_2max_ for 3 weeks. In that study, the authors also suggested that low-intensity exercise favors fat oxidation and is more feasible and acceptable than high-intensity exercise for obese adolescents.

### 3.10. Regular Low-Intensity Exercise and BP

Clinical data have provided an association between oxidative stress and hypertension [66]. Ye et al. [67] suggested that reactive oxygen species (ROS) may elevate BP by stimulating the sympathetic nervous system (SNS). Because NO exerts tonic inhibition on central SNS activity, increased ROS production may trigger the SNS through oxidation/inactivation of NO. Hua et al. [68] quantified the effects of a 12-week home-based low-intensity walking exercise program in hypertensive men and women and showed a training effect in which SBP, DBP, and the R-R interval decreased significantly, and spontaneous baroreflex sensitivity increased significantly in the exercising group.

In this study, the decline in BP was not statistically significant. SBP and DBP decreased by approximately 2 and 0.5 mmHg in the continuous walking group, respectively, whereas in the intermittent walking group, SBP and DBP decreased by approximately 4 and 0.5 mmHg, respectively. A study by Lu et al. [26] conducted in adults with hypertension and prehypertension revealed that regular sessions of slow walking for 50–60 min/day at 5–7 times/week significantly lowered BP and HR. In that study, the authors suggested that *β*-endorphin could act as a buffer against exercise-related sympathetic excitation and cardiovascular overload, which may play a role in pressure-lowering and negative chronotropic effects. Motoyama et al. [69] observed that resting SBP (−15 mmHg), MAP (−11 mmHg), and DBP (−9 mmHg) decreased significantly following 3 months of low-intensity treadmill exercise for 30 min, 3–6 times a week in elderly hypertensive patients. The slight decrease in SBP and DBP without major significance in our study compared with previous reports may be due to a difference in duration, frequency of exercise, and training period. Nevertheless, a greater reduction in SBP in the intermittent walking group resulted in decreased PP in this group after exercise program participation. Moreover, with a significantly lower resting HR and greater reduction in SBP in the intermittent walking group, RPP was expressed to be lower in participants who partook in intermittent walking. Normally, BP control is also associated with other influencing factors, including baroreflex sensitivity, endothelial function, and the renin-angiotensin system [70]. It seems that those factors need to be correspondingly improved for BP lowering since they appear to be intensity- and mode-dependent [71].

Notably, it has been estimated that a 2 mmHg decrease in SBP would result in a 6% overall reduction in mortality due to stroke, a 4% reduction in mortality due to coronary heart disease, and a 3 to 4% reduction in all-cause mortality [72]. Accordingly, our findings emphasize the effects of long-term regular walking as exercise therapy in older people with hypertension. Furthermore, the results encourage the Guideline Recommendations of the American College of Cardiology and American Heart Association in that regular exercise should be prescribed for the prevention and management of hypertension [73].

### 3.11. Study Limitations

This study has certain limitations. Most of the participants received concomitant medications, such as calcium channel blockers, angiotensin receptor blockers, angiotensin converting enzyme inhibitors, beta-blockers, or diuretics, before and during the exercise program. Some findings may be partly affected by those treatments, although there was no change in medications administered during the study. In addition, few studies have evaluated the effects of long-term regular low-intensity exercise on BP and the relevant factors. Also, no study has compared continuous to intermittent low-intensity exercise in older populations with hypertension. Consequently, our discussion is based on closely related evidence. This study program was designed as a home-based exercise that afforded participants flexibility in terms of performing the exercise. Nevertheless, good adherence to and compliance with the exercise programs was reported.

## 4. Conclusions

This study provides evidence that practicing long-term regular continuous and intermittent walking may hold potential for increasing antioxidant and decreasing lipid peroxidation, thus alleviating oxidative stress in older participants with hypertension. Moreover, the participants practicing continuous walking exhibited greater improvement in metabolic profile, while the participants practicing intermittent walking showed greater improvement in cardiac autonomic function.

## Figures and Tables

**Table 1:** Physical and physiological characteristics of participants before and after the exercise program.

	CON group (*n* = 22)		INT group (*n* = 21)		*p* value
	Before	After	Before	After	
Age (yrs)	70.95 ± 5.00	71.20 ± 5.00	70.86 ± 6.51	71.11 ± 6.51	0.956
Gender (M/F) (%)	8/14 (36/64)	8/14 (36/64)	2/19 (10/90)	2/19 (10/90)	0.037
Height (m)	1.57 ± 0.08	1.57 ± 0.08	1.55 ± 0.07	1.55 ± 0.07	0.524
Body mass (kg)	56.65 ± 10.72	56.66 ± 10.57	59.33 ± 8.11	57.07 ± 10.66	0.592
BMI (kg/m^2^)	22.97 ± 3.54	22.93 ± 3.59	24.55 ± 2.66	22.95 ± 3.53	0.369
Body fat (%)	29.71 ± 8.09	29.68 ± 8.32	36.65 ± 6.36	30.84 ± 7.59^*∗*^	0.334
Fat mass (kg)	17.03 ± 6.25	17.04 ± 6.33	21.83 ± 5.13	17.76 ± 5.92^*∗*^	0.300
Fat-free mass (%)	70.29 ± 8.11	70.33 ± 8.33	63.29 ± 6.31	69.17 ± 7.60^*∗*^	0.317
Fat-free mass (kg)	39.61 ± 7.79	39.62 ± 7.69	37.46 ± 5.92	39.31 ± 7.84	0.517
Body water (%)	51.77 ± 6.08	51.79 ± 6.21	46.59 ± 4.73	50.90 ± 5.64^*∗*^	0.328
Water mass (kg)	29.18 ± 5.77	29.18 ± 5.69	27.59 ± 4.45	28.93 ± 5.80	0.541
Protein mass (kg)	7.74 ± 1.55	7.73 ± 1.53	7.24 ± 1.16	7.67 ± 1.55	0.434
Mineral mass (kg)	2.70 ± 0.49	2.71 ± 0.48	2.68 ± 0.40	2.71 ± 0.49	0.873
Visceral fat level	6.91 ± 2.49	6.95 ± 2.50	8.71 ± 1.82	7.24 ± 2.28^*∗*^	0.176
Waist circumference (cm)	82.30 ± 11.52	81.07 ± 11.52	85.54 ± 7.77	81.10 ± 11.60	0.473
Hip circumference (cm)	94.01 ± 9.04	92.72 ± 7.89^*∗*^	98.50 ± 5.78	93.33 ± 7.61^*∗*^	0.333
W/H ratio	0.88 ± 0.06	0.88 ± 0.06	0.89 ± 0.06	0.88 ± 0.05	0.989

Data are mean ± SD. CON, continuous walking; INT, intermittent walking; BMI, body mass index; W/H, waist to hip circumference ratio. ^*∗*^*p* < 0.05, significantly different from before exercise program (two-way ANOVA).

**Table 2:** Metabolic profile of participants before and after the exercise program.

	CON group (*n* = 22)		INT group (*n* = 21)		*p* value
	Before	After	Before	After	
Glucose (mg/dL)	93.14 ± 8.62	93.09 ± 19.79	94.14 ± 14.57	89.00 ± 11.38^*∗*^	0.188
TG (mg/dL)	109.14 ± 39.31	86.05 ± 34.16^*∗*^	109.90 ± 44.72	110.67 ± 50.38^#^	0.017^#^
LDLC (mg/dL)	127.68 ± 31.69	121.36 ± 33.07	146.00 ± 38.56	144.57 ± 29.46	0.106
HDLC (mg/dL)	48.82 ± 7.77	52.18 ± 6.46^*∗*^	54.48 ± 15.00	54.95 ± 15.57	0.336
TC (mg/dL)	198.36 ± 32.76	190.73 ± 34.78	222.57 ± 39.48	221.71 ± 35.35	0.080
TC/HDLC ratio	4.14 ± 0.85	3.70 ± 0.78^*∗*^	4.35 ± 1.24	4.29 ± 1.21^#^	0.048^#^
AI	3.14 ± 0.85	2.70 ± 0.78^*∗*^	3.35 ± 1.24	3.29 ± 1.21^#^	0.048^#^

Data are mean ± SD. CON, continuous walking; INT, intermittent walking; TG, triglyceride; LDLC, low-density lipoprotein cholesterol; HDLC, high-density lipoprotein cholesterol; TC, total cholesterol; AI, atherosclerogenic index. ^*∗*^*p* < 0.05, significantly different from before exercise program (two-way ANOVA). ^#^*p* < 0.05, significantly different from the CON group (two-way ANOVA).

**Table 3:** Heart rate, heart rate variability, and blood pressure of participants before and after the exercise program.

	CON group (*n* = 22)		INT group (*n* = 21)		*p* value
	Before	After	Before	After	
HR (beats/min)	63.08 ± 8.87	63.91 ± 9.38	63.83 ± 5.92	60.44 ± 6.38^*∗*^^#^	0.014^#^
SDNN (ms)	47.41 ± 21.54	36.58 ± 15.40^*∗*^	47.96 ± 34.84	49.85 ± 26.70^#^	0.016^#^
RMSSD (ms)	49.60 ± 37.05	34.02 ± 24.97^*∗*^	47.08 ± 59.92	51.59 ± 44.77^#^	0.018^#^
TP (ms^2^)	2579.75 ± 2405.32	1577.64 ± 1710.37	3233.71 ± 6002.76	2891.53 ± 3578.45	0.086
VLF power (ms^2^)	1218.70 ± 1487.64	765.12 ± 685.81	1038.63 ± 780.51	943.27 ± 863.62	0.358
LF power (ms^2^)	256.73 ± 289.64	204.92 ± 204.83	524.56 ± 836.79	442.87 ± 600.78	0.237
LFnu power	28.91 ± 18.16	37.20 ± 20.52	41.97 ± 16.65	30.55 ± 13.46^*∗*^^#^	0.043^#^
HF power (ms^2^)	787.38 ± 806.58	481.68 ± 993.05	1046.87 ± 2586.93	1135.71 ± 2138.87	0.081
HFnu power	55.43 ± 15.60	48.81 ± 16.94	47.45 ± 9.83	54.92 ± 13.27	0.103
LF/HF ratio	0.65 ± 0.63	1.21 ± 1.68	0.94 ± 0.51	0.67 ± 0.48	0.160
SBP (mmHg)	127.50 ± 15.35	125.82 ± 12.28	129.97 ± 14.84	125.79 ± 15.03	0.626
DBP (mmHg)	76.79 ± 8.42	76.27 ± 6.71	74.87 ± 7.00	74.11 ± 7.38	0.550
PP (mmHg)	50.71 ± 12.20	49.55 ± 11.22	55.10 ± 11.06	51.68 ± 11.94^*∗*^	0.646
MAP (mmHg)	93.69 ± 9.63	92.79 ± 7.23	93.24 ± 8.89	91.34 ± 8.94	0.531
RPP (mmHgbpm)	8058.96 ± 1609.64	8058.35 ± 1499.66	8250.85 ± 827.37	7580.20 ± 1043.28^*∗*^^#^	0.049^#^

Data are mean ± SD. CON, continuous walking; INT, intermittent walking; HR, heart rate; SDNN, standard deviation of the NN intervals; RMSSD, root mean square of successive differences; TP, total power; VLF, very low frequency; LF, low frequency; HF, high frequency; SBP, systolic blood pressure; DBP, diastolic blood pressure; PP, pulse pressure; MAP, mean arterial pressure; RPP, rate-pressure product. ^*∗*^*p* < 0.05, significantly different from before exercise program (two-way ANOVA). ^#^*p* < 0.05, significantly different from CON group (two-way ANOVA).

## Data Availability

No data were used to support this study.
